# Personal Learning Environments that Facilitate Socio-Educational Integration of Unaccompanied Foreign Minors

**DOI:** 10.3390/ijerph17145012

**Published:** 2020-07-13

**Authors:** María del Carmen Olmos-Gómez, María Tomé-Fernández, Eva María Olmedo-Moreno

**Affiliations:** 1 Department of Research Methods and Diagnosis in Education, Faculty of Education and Sport Science, University of Granada, 52071 Melilla, Spain; practicas_faedumel@ugr.es; 2 Department of Research Methods and Diagnosis in Education, Faculty of Education Science, University of Granada, 28071 Granada, Spain; emolmedo@ugr.es

**Keywords:** personal learning environments (PLE), unaccompanied foreign minors (UFM) adolescent adjustment, adolescent competence social factors

## Abstract

The aim of the present study was to predict the variables that facilitate integration of unaccompanied foreign minors (UFM) and to develop personal learning environment (PLE) questionnaire dimensions with respect to social integration of UFM. *Methods:* A social study that was descriptive in nature was conducted with a quantitative empirical-analytical focus. *Results:* Results from discriminant function analysis indicate that 86% of group membership was correctly classified from gender alone, with female learning environments leading to greater future success. *Conclusions:* It can be concluded that the predictive results possess methodological coherence. Thus, from them we can propose possible development strategies, particularly targeting males, in order to improve learning and promote social integration. According to the results obtained, improvement of learning strategies and strengthening of the very learning environments, demands new policies to be established which promote emotional improvement and better futures for UFM, especially males.

## 1. Introduction

Constant migratory movement towards cities that offer better basic opportunities, is a fact that characterises the majority of developed countries of the 21st century [1].

In recent years, refugees of the Syrian civil war, emigrants from the African Horn, and Iraqi citizens fleeing from a country terrorised by the Jihadist group ISIS make up a list of immigrants who, for a number of decades now, have been attracted by European economic development and social welfare [2]. As a consequence, many families arrive in full to the continent. However, there is also a large number of minors who, for different reasons, enter this territory without any companion or legal guardian [3].

There are currently shocking images on the television and numerous new stories in the written press about minors who try to reach some member countries, crossing the sea in unstable boats, or by land in hidden sections of cars and lorries. Thus, as a result of immigration it is normal in European border cities for children to live together in street ghettos [4]. In European legislation these individuals are denominated as unaccompanied foreign minors (UFM) (European Council of the 26 June 1997) [5]. 

Various studies describe the psychosocial characteristics of minors who survive in the street [6,7,8,9,10,11,12,13,14]. These concur in highlighting that many suffer emotional problems which often translate to drug and alcohol abuse, and behavioural problems which provoke conflict, even amongst themselves. In confronting this situation, institutional care centres are seen as the best alternative to the streets, offering these children a safe place free from violence. Here these children develop a good educational level which will improve as they become more socially integrated [15]. However, different studies also highlight the reduction in institutional resources apportioned to these minors once they reach adolescence [16,17,18,19,20].

The importance of education as a key element in the social and educational integration of vulnerable minors [21] forms the basis of the present research. The research seeks to better understand the socio-educational integration strategies in place within personal learning environments (PLE) and the conditions favourable to education enjoyed by unaccompanied minors found residing in Spanish institutional centres. 

Spain is one of the border countries of the European Union, where 3261 unaccompanied minors were recorded according to data from the Spanish Ministry of the Interior in 2013. This number would probably be even higher if it had been possible to register minors living on the streets who had never resided in institutional centres [22].

In the same way as with what happens in the other member countries, Spain has a protocol for good practice which states that once in a country, minors become the responsibility of the communities or autonomous cities who regularise their legal status [23]. This assures that unaccompanied minors begin to reside in institutional care centres within the host cities. Here they receive food, a place to sleep, health assistance, psychosocial support and are guaranteed a place to attend nearby academic institutions.

Although international studies exist that have sought to better understand the important aspects of these children’s vital development and the policies available to support their integration [24,25,26,27,28,29] research into the influence of educational aspects on the social integration of these minors is scarce. Only Wallin and Ahlström [25] have embraced the educational theme as one of the key cogs in the integration process of UFM, but they do not specify the most influential socio-educational strategies for this integration. This is also the same in Spain, where studies focusing on socio-educational strategies for these minors are non-existent. From this perspective, the importance of the study is clear.

To better understand the socio-educational strategies used by unaccompanied minors who live in institutional centres, the personal learning environments (PLE) of these centres will be analysed. According to Chatti, Augustiawan, Jarke and Specht [30] this environment is all that is employed by a person, consciously or otherwise, to learn. It is therefore a new way of accessing learning, where the acquisition of information is not limited by either space or time. It is also where new norms for relating oneself with other users through computer/mobile applications and social networks come into play [31,32].

Castañeda and Adell [33] differentiate three types of socio-educational strategies which make up the PLE. Reading strategies correspond to the sources of access to information, which may or may not use new technologies (Newsletters, impact blogs, network video channels, quick reads, summarising textbooks, attendance at conferences, viewing of audio-visuals, etc.). Reflexion strategies are reliant upon environments where information can be transformed and reflected upon (blog, publication of videos, the wall of social networks, notebooks, class diary, etc.). Finally, relational strategies are linked to the virtual or physical spaces in which students associate for learning (social networks, applications, the school classroom itself, etc.).

It is generally accepted that PLE’s concurs with regards to the main conclusions reported. These state that students who use the aforementioned strategies will become autonomous beings and demonstrate a greater capacity for decision making [34,35,36,37,38,39,40]. Strategies will also facilitate their academic and social integration as a consequence of being engaged in different types of learning communities [41].

When unaccompanied minors arrive to institutional welcome centres they normally live with children of the same age and race who have lived through similar traumatic experiences. This does not facilitate their social integration, as they take refuge in their peers and have a strong sense of belonging to this specific group [42] The learning communities that are formed when putting the strategies composed by PLE’s into place, can reduce the social isolation that is often present in these minors [43,44]. This, together with the fact that the majority of minors received by Spain belong to the Islamic religion coming from the northern zone of Maghreb, which is typified by some social values [45,46,47] which may be different to those promoted by developed democracies [48], makes it increasingly necessary to promote socio-educational strategies which help the social integration of minors.

Without scaremongering, we must not forget that various studies have associated the formation of ghettos within Muslim populations with their radicalisation [49,50]. This leads us once more to extol the benefits of learning communities which arise in the PLE and are formed through the use of socio-educational strategies. It is here where unaccompanied minors are able to share experiences, reproduce information, work through common themes and ultimately, interact with other children from the cities where they live [51]. In this way they can start to form a European identity that enables them to become young democratic citizens of the future, who enjoy civil commitment [52].

**Figure 1:**
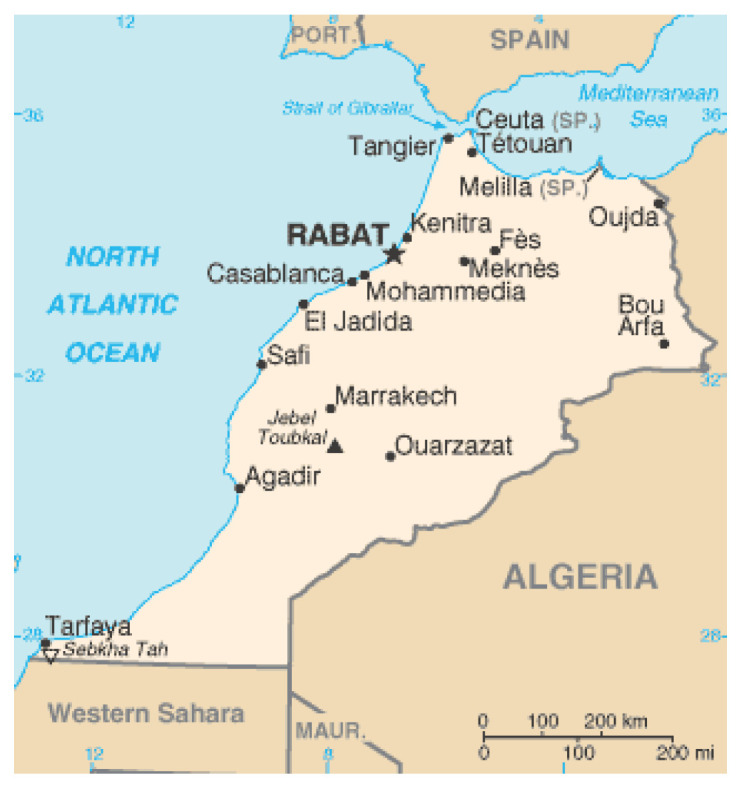
Geographic situation of the city of Melilla. ©Wikimedia.org.

Melilla is a Spanish city that is found in the north of Africa. Its border adjoins with the country of Morocco, with the majority of UFM who end up taken in at the different institutional centres of the city hailing from here (Figure 1).

According to the Spanish Institute of National Statistics (2017), despite being one of the smallest cities in longitude, it has taken in one fifth of the country’s overall UFM population since the year 2014. In this year specifically, welcome centres in Melilla received 654 UFM. This quantity has increased progressively over the following years, with 759 UFM being registered in the year 2015, 999 in the year 2016 and 724 in the first nine months of the year 2017.

As has been previously mentioned, the importance of the PLE in the socio-educational integration of UFM, lies in the use of strategies that promote the creation of learning communities in which UFM can interact with students from the city and with other foreign students.

These aforementioned communities are characterised by the presence of a group of people with common objectives [53] who maintain a quality relationship within a determined space [54], that can also be virtual in nature, such as Brailas et al. [55].

In addition to taking into account the cultural diversity of learning communities formed by foreign minors and native students, appropriate management of human resources within the relevant learning environments also has a special significance [56]. The purpose of this being that socio-educational integration becomes a reality.

According to Sánchez-Medina, Gómez-Stern and Martínez-Lozano [57], a combination of two situations is produced by the integration of immigrants into learning communities. On the one hand, the immigrant adapts to the characteristics of the welcome centre, with regards to habits, values and norms. On the other hand, they maintain the cultural practices and values that are allowed by members of the community. In this case, these social interactions strengthen the acquisition of social abilities which will help foreign minors later on to efficiently manage their social relationships and achieve a positive coexistence [58].

From another perspective, an important aspect to take into account in the present study is that the first learning communities to be formed by UFM, will be shaped alongside other foreign students. These is due to the fact that in some welcome centres both groups live together (unaccompanied and those with parents residing in the city). In the case of the latter, the parents of the foreign minor lives in Melilla, sometimes in an irregular way, without being able to take economic responsibility for their children but maintaining regular scheduled visits to the institution.

Socio-cultural theory on the topic, describes the importance to minors of having social interaction and the crucial role of the adults closest to them [59]. The fact that some foreign minors maintain a systematic relationship with some of their parents could favour experiences that promote the learning and development of these children [60]. Just as has been affirmed by some studies, family participation in the student’s learning community positively influences their academic performance [56,61,62,63]. Indeed, in some cases, parents can improve the cultural adaptation of their children, favouring the learning of certain circumstances that will substantially improve their integration [64].

Ultimately, the creation of learning communities of UFM with other students, whether they are foreign or not, will favour both virtual and real situations which will help them to get out of their comfort zone and to deal with situations, which can sometimes be hostile, with people of different beliefs, values and attitudes [65]. The constant interaction produced within learning communities, will promote adaptation of UFM to the culture of the host community, with them being socially integrated to a greater or lesser extent [66].

The present study aims, is to examine whether gender is a predictive and discriminate variable when considering the personal learning environments of UFM?

In agreement with the prior analysis, the objectives of the present study are as follows:

- To better understand the PLE’S that favour social integration of UFM and examine whether gender is a discriminant factor in the use of these PLE’s.
- To determine the likelihood of future associations between the use of PLE’s and future development.

The present study takes a quantitative focus and adopts an empirical-analytical social research method that is descriptive in nature [67].

## 2. Methods

### 2.1. Participants

The sampling approach developed was non-probabilistic and ad hoc, and depended upon accessibility of participants. In this respect, 356 UFMs participated with an average age of 14.22 years (SD = 2.968) aged between 8 and 17 years (8–10 = 5.05% (*n* = 18); 11–13 = 31.45% (*n* =112); 14–16 = 36.8% (*n* = 131); 17 = 26.7% (*n* = 95)), from the autonomous city of Melilla. As a function of gender, 260 were male (73.1%) and 95 females (26.9%). With respect to the stage of education, 29.5% were studying primary education, 19.2% secondary education, 0.6% a bachelor’s degree, and 13.5% vocational training courses.

From the overall sample of 356 participants, most came from Morocco (73.7%), followed by non-specified locations (11.5%), with Fes, Rabat and Germany providing one or two participants, Guinea providing 2.6% of the sample and France and Barcelona each providing one participant, respectively.

### 2.2. Instrument

The socio-educational integration strategies questionnaire (SINQ) of Olmedo et al. [68], was used to evaluate socio-educational integration strategies of UFM through analysis of their personal learning environments and social values. The questionnaire is formed by 40 items, with a five-point Likert response scale, where 1 = strongly disagree and 5 = strongly agree. It evaluates four dimensions: Self-concept, planning and management, use of resources and tools, and communication and social interaction. The four-factor rotation was conducted, with the following Bartlett statistics being obtained, (4418.7 (df = 780; *p* = 0.000010)). As for the values obtained in the KMO (Kaiser–Meyer–Olkin test), the value of 0.814 acquired reflects acceptable adjustment suggesting that the data can be submitted to factorial analysis. This provides an index that determines sampling adequacy and whether the proportion of variance among variables measured from a population might share a common variance. Through this, it was found that 44% of total variance can be explained through four factors, the GFI (goodness of fit index) was 0.95 and the RMSR was 0.055, with these values indicating good adjustment of the items [69].

### 2.3. Process

#### Data Collection

Data collection counted on the voluntary participation of foreign minors being sheltered in children’s centres, specifically in the Centres “La Purísima” (the Purest Centre) and “La Divina Infantita” (the Divine Infant Centre). Permission was received prior to commencing from the autonomous city of Melilla to access the centres. In developing the present study, questionnaires were administered outside of the normal hours of the centre so as not to interfere with the timetable already planned by the centres. Once agreement from the director of the centre and the Social Welfare Council of the autonomous city of Melilla was obtained, questionnaires were administered on paper and in Spanish, in a group environment to small groups of UFM in order to facilitate better understanding and enable completion of the questionnaire with the help of a translator Monitors and social educators were present to lend support and facilitate understanding of questions. It was guaranteed that all of the collected information would be used only for scientific research purposes and that anonymity of participants would be maintained throughout. Participants were not informed about the purpose of the study in order to avoid insincere responses being given and to reduce the possible effects of social desirability.

## 3. Results

### Data Analysis

Table A1 (Appendix A) shows that all of the response categories were chosen by a percentage of students and that their standard deviations are all superior to 1.346, this indicates that the items were able to discriminate to an acceptable level. The “corrected item-total correlation” (ri-t) is positive for all items, with values between 0.185 and 0.751. This indicates that all items contribute to the measurement performed by the overall test and also run in the same direction. Table 1 shows results grouped according to factors, with the large effects sizes relating to the PLE standing out. The sample size or proportion of explained variance (ANOVA) indicated that, with respect to the self-concept of the learning process (SLP) factor, more than 59% (η^2^ = 0.59) of the differences found can be attributed to the effect of gender. In the same way, results obtained large effect sizes with respect to gender for the factors of planning and management of learning (PML) and use of resources and tools (URT), these being 15% and 20%, respectively. Likewise, with respect to Eta-squared, in the Table 2 shows that it is highlighted that the variables that reach significance are as follows: I am able to maintain a conversation with other people in a foreign language (Spanish) as much for academic purposes as for social ones (0.54); I am able to maintain a conversation I am able to write a digital document such as the opening of a blog or an email in my own language (0.83) and I am able to define future short and long-term goals, and plan the steps to reach them (0.55), this is related to the discriminant analysis that will now be presented in the results section, however, all results higher than 0.14 are also shown because eta-squared values > 0.14 are typically interpreted as large.

Once the psychological properties of the evaluation instrument to be employed were guaranteed, that is, its reliability and validity, a discriminant analysis was conducted of the variables whilst controlling for the extraneous variable of gender. Discriminant analysis offers an appropriate statistical test for selecting the independent or predictive variables that differentiate groups. It also enables examination how many of these variables should be included to reach the most accurate classification possible. Further, this analysis permits quantification of the discriminative power of a subject or an object belonging to a particular group. For this reason, whilst also being a classification test, this technique is considered a test of dependence. In fact, its approach is similar to logistic regression, with the only difference being that it only admits quantitative variables. When a population that is considered to be immigrant or non-native speaks, we wish to establish the discriminatory factors influencing perceptions based on ethnic origin, nationality and, even, culture (especially, religion). Nonetheless, such differential experiences tend to be related with other observed characteristics: Sex (female), age (older individuals) and economic situation (low socioeconomic status). For this reason, we have conducted a discriminant analysis in order to be able to establish the explanatory and discriminatory power of the characteristics that differentiate UFM, according to their PLE and as a function of gender [70,71].

Quantitative data were analysed according to descriptive statistics and estimations of internal consistency, this was done using SPSS 24.0 (International Business Machines Corporation (IBM), Chicago, IL, USA, 2019).

356 participants belonging to children’s centres in the autonomous city of Melilla participated in the present study. The objective of the study was to identify the independent variables to have the greatest discriminatory and predictive power for classifying participants as a function of gender.

In this case, it was desirable to predict the probability of PLE use according to gender. To this end gender was incorporated and the variables of tolerance to and ease of use of the questionnaire were to be examined.

The descriptive statistics used for this were the Box’s M test, which contrasts the equality of covariance matrixes, and as is reflected in Table 3, significance testing, which confirms whether one group is more variable than another by examining whether the variance-covariance matrixes are different.

Tests the null hypothesis that the population covariance matrices are equal.

Summary of findings from the canonical discriminant analysis.

The results obtained and presented in Table 4, (eigenvalue 2.849, canonical correlation 0.860) enable it to be concluded that a single discriminant function exists that is statistically significant (sig. 0.000), thus participants can be classified into two groups. The Wilks λ value (0.260) gives rise to a second conclusion that not all variables are shown to discriminate when predicting group membership and thus the discriminant function. Thus, the influence of each one of the variables on the discriminant function obtained will be studied.

The first 1 canonical discriminant functions have been used in the analysis.

Table 5 presents the centroid matrices where group means for the functions are obtained. In our study, these values are different, negative values in the group classified as male illustrate a negative influence of the selected variables, which shows that, within the female group, the higher the value the greater influence it has.

**Figure 2:**
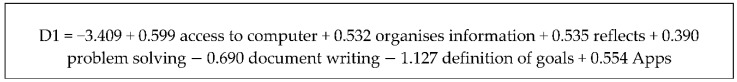
Identification of the resultant discriminant function.

The discriminant functions based on the standardised coefficients shown in Table 4 identify those variables with greatest weight in the predictive model and enables identification of the resultant discriminant function (Figure 2 and Table 6).

In our case (Table 6), we observe that 86% of observations were correctly classified from data on personal learning environments and gender. Female UFMs present greater aptitude with respect to personal learning environments. Thus, according to the results, males achieve less success as a function of their personal learning environment.

## 4. Discussion

Firstly, the psychometric characteristics of the instrument employed, this being the socio-educational integration strategies questionnaire (SINQ) of Olmedo et al. [68] which analyses the personal learning environments and social values of UFM, are found to be adequate with respect to reliability and validity. To this end, adequate psychometric properties were found showing satisfactory fit, as well as valid reliability indices as indicated by George and Mallery [72]. In summary, it can be noted that the results indicate the existence of four factors which form the instrument: Self-concept, planning and management, use of resources and tools, and communication and social interaction. The ten experts who participated in the validation of the instrument fulfilled specific quality criteria and were highly experienced in the field of social pedagogy. As has been previously indicated, the instrument showed adequate internal consistency at both a general level and with regards to each of its individual dimensions. This supports its application within any context involving UFM. In this way, the identified dimensions guide us towards a deeper knowledge of diverse aspects of the social integration of UFM groups and their personal learning environments as being crucial for inclusion of this population group [69].

The SINQ questionnaire applied to the PLE and social values of UFM corresponds to the study conducted by Castañeda and Adell [33] where three types of socio-educational strategies were differentiated which compose the PLE. Reading strategies: Correspond to sources of access to information which may or may not include new technologies (newsletters, impact blogs, network video channels, quick reads, textbook summaries, attendance to conferences, viewing audio-visuals, etc.). These refer to our first capacity factor, where the incorporated items are directly related with this strategy. Reflexion strategies: Incumbent upon the environments where information can be transformed and reflected upon (blog, publication of videos, the wall of social networks, notebooks, class diary, etc.), this corresponds to our second factor for engagement. Finally, relational strategies: These types of strategies are linked to the virtual or physical spaces with which students associate in order to learn (social networks, applications, the classroom itself, etc.) and provide our third factor for the development of PLE. The need therefore emerges to work towards the PLE factors (self-concept, management and use of resources) through improvement of the levels of communication and social interaction, keeping in mind that the social actors should learn to see this population within their social network whilst recognising the double belonging of being both “from here” and from their country of origin [73]. This will provide the foundation of an inter-cultural education capable of achieving satisfactory levels of communication and integration [69,74].

Secondly, the results of the summary table of the classification of participants according to the outcomes of the discriminant function obtained, should be highlighted. Specifically, they underline that through realisation of the discriminant function obtained, 86.0% of participants were classified correctly from their gender. This tells us that the learning environments of females are those that will be more successful in the future. The discriminant analysis conducted established the explanatory and discriminatory power of the characteristics differentiating UFM. We then proceeded to conduct a study of series of independent variables, including gender as a discrimination variable. Through examination of the description of the degree of existing relationships between the set of variables, the analysed characteristics identify gender as a discriminatory variable. As a main output, a classification rule will be obtained which can be used to predict future events as a function of PLE’s [54,70,71]. This promotes the inclination to think that girls who develop and use learning strategies will become autonomous beings with a greater decision making capacity [34,35,36,37,38,39,40] besides also facilitating their academic and social integration as a consequence of participation in different types of learning communities [41]. With regards to the relationship between welfare provision and immigration control, welfare provision should exist at a local level where it can contribute to immigration control. Examples of this may include, when the objective is to facilitate the resolution of migration status or to reconnect with the country of origin, or when access to a refuge is conditional on compliance with return processes. Despite this, we see that social care provision for irregular migrants can also have the purpose of fulfilling alternative policy objectives, such as public health or the protection of children [75]. To this end, the results obtained enable prediction of future expectations for the girls studied and suggest how strategies for children can be optimised.

## 5. Conclusions

In conclusion, the present research was conducted with a fairly representative sample of UFM from the autonomous city of Melilla, which enabled us to establish predictive results with methodological coherence and to suggest possible development strategies to improve the learning of these children and promote their social integration. Ninety percent of UFM have their hopes set on arriving to a host destination outside of the border city of Melilla. Their aspirations centre on two fundamental aspects, to get out of Melilla in search of new opportunities in the mainland and then later on in Europe, and to get residency papers. As a result, they undertake numerous interrupted journeys during which migrants use mobility to ensure their basic needs are met and to avoid the attempts of migration control. However, this increase in mobility can aggravate emotional instability [76]. Improvement of learning strategies and strengthening of the learning environments in which they are present, according to the results obtained, endorse the establishment of new policies to promote emotional improvement and a better future for UFM. Documentation relating to the protocol for the study conducted by Castillo [77] was requested following a three-month continuous stay at the care residence, with the Ministry of Social Welfare requesting the initial residency card and identification. Nonetheless, this same study detected that many minors who had resided for more than 2 years at the centre for minors (named CERM) turned 18 before applying for a renewal of their residency. Some minors who had been at the centre for a year or less, also experienced difficulties in continuing with the submission of documentation after reaching adult age because they had not completed the documentation process [77]. This occurs because the period of time at which documentation must be completed changes from 3 to 6 months. This situation leaves many of these young people undocumented and means that they are unable to return to their country. Instead, they must stay in the city of Melilla wandering the streets, hoping for their situation to be resolved and, in some cases leading to an opening for a quick expulsion. This situation directly affects the “day to day” of the youth centre given that many of the minors hang around the centre, leading to thefts and aggression against other minors [6,7,8,9,10,11,12,13,14,77]. For this reason, it is important to better understand the socio-educational integration strategies administered through the personal learning environments (PLE), of unaccompanied male minors [25]. Such approaches offer a way to improve the future development of these minors and, in this way, reinforce actions to improve integration whilst also providing us a useful research perspective [21,24,25,26,27,28,29,78,79]. There are different training experiences for counsellors and mentoring peer support which, although difficult to implement, use the service-learning methodology. This is a methodology which can facilitate the development of critical awareness and social commitment of these minors, addition play a fundamental role in this process of social integration [80,81].

It would be interesting for future research to broaden the sample to include UFM from other similar cities or even to increase the range of welcome centres included from the Spanish mainland or across Europe. Further, it would be useful to employ different evaluation instruments, which should be both quantitative (other complementary questionnaires) and qualitative (for example through interviews). The importance of the study theme and the expansive framework used opens up research opportunities and promotes the development of follow-up studies, where the present work provides a point of reference.

Amongst the limitations identified in the present study was the difficulty of accessing the sample, though the time required to collect data was found to be reliable. From this it is concluded that the results of the study of the PLE in UFM populations as a function of the extraneous factor of gender, are revealing and are hugely important to take into consideration in future research. For the majority of UFM, one of the main difficulties is communication. Del Sol-Flórez [82] indicates that the commitment UFM put into learning Spanish is an important factor in inclusion.

## Figures and Tables

**Table 1:** Mean, standard deviation (SD) and Eta-squared (η2) obtained after administration of the socio-educational integration strategies questionnaire (SINQ).

Factors	Gender	Mean	SD	η^2^
Self-concept of the learning process (SLP)	Male	3.59	1.74	0.059
	Female	3.04	1.87	
Planning and management of learning (PML)	Male	3.84	1.56	0.015
	Female	3.76	1.63	
Use of resources and tools (URT)	Male	3.70	1.52	0.020
	Female	3.62	1.44	
Communication and social interaction (CSI)	Male	3.68	0.89	0.004
	Female	3.86	0.92	

**Table 2:** Summary of the most representative data with regards to eta-squared y mean, standard deviation and discrimination index obtained after administration of the socio-educational integration strategies questionnaire (SINQ).

Items	Mean	SD	R_i-t_	η2
4. I am able to reflect before selecting the most appropriate digital tools for my learning.	3.55	1.504	0.487	0.042
7. When I am presented with a problem, I evaluate its importance, analyse its causes and dedicate time to resolve it.	3.52	1.487	0.372	0.027
10. I am able to maintain a conversation with other people in a foreign language (Spanish), as much for academic purposes as social ones.	3.29	1.389	0.485	0.054
11. I interact with other people for academic purposes, using tools from my personal learning environment (PLE) such as chats and forums.	2.91	1.469	0.463	0.035
13. I share information that I know or have constructed, using tools from my PLE for this such as apps, chats, YouTube and forums.	2.61	1.563	0.280	0.016
15. I am able to write a digital document such as the opening of a blog or email in my own language.	3.07	1.591	0.416	0.083
17. The fact of being in an unusual situation affects my participation and access to educational resources.	3.12	1.549	0.254	0.018
20. Lack of social support affects my participation in activities in my community or reduces my access to resources.	3.52	1.567	0.494	0.029
22. My opinions are valued in my community.	3.29	1.492	0.626	0.030
25. I would be able to find someone who will help me to feel better.	3.65	1.414	0.227	0.017
28. I identify with my community.	3.59	1.266	0.319	0.016
31. When working or communicating with other people, I listen to the different points of view and I respect them.	3.78	1.314	0.514	0.016
34. I like to embark upon new projects.	3.72	1.278	0.694	0.038
35. I am able to define short and long-term goals, and to plan the steps to reach them.	3.73	1.427	0.742	0.055
37. I engage in socio-recreational activity in my community.	3.54	1.142	0.703	0.020

Response percentage for each scale option, mean, standard deviation and discrimination index obtained after administration of the socio-educational integration strategies questionnaire (SINQ), obtained from the present sample (*n* = 356): Mean; SD.

**Table 3:** Results from statistical testing.

**Box’s M**		55.239
**F**	Approx.	1.566
	df1	28
	df2	2.670
	***p***	0.030

df = degree freedom.

**Table 4:** Eigenvalues.

Function	Eigenvalue	% of Variance	Accumulated %	Canonical Correlation
1	2.849	100.0	100.0	0.860

**Table 5:** Structure matrix.

Structure Matrix PLE	Function
	1
15. I am capable of writing a digital document such as the opening to a blog or email in my own language.	−0.273
7. When I am presented with a problem, I evaluate its importance, analyse its causes and dedicate time to resolve it.	0.232
23. I am able to recognise when I make a mistake and I try to correct it in the most satisfactory way possible.	−0.225
12. I interact with others for social purposes (to meet new people, to form friendships, to inform myself about new trends, etc.), using tools from my PLE such as chats, forums, social networks, blogs, wikis, etc.	0.224
14. That seen, heard and read online, in chats and in digital libraries, etc. has been able to modify, clarify and/or broaden a concept seen in class.	0.217
26. I know how to say NO when I believe that I have to.	0.208
38. My PLE (digital tools) improves my autonomous working and helps me in my activities as a student and to escape from daily life.	−0.188
16. I am able to write a digital document such as the opening of a blog or email in a foreign language (Spain).	−0.185
4. I am able to reflect before selecting the most appropriate digital tools (apps, chats, YouTube, forums, social networks, blogs, wikis …) for my learning.	0.185
1. I have access to computers, tablets, smartphone, etc.	0.181
20. Lack of social support affects my participation in community activities or my access to resources.	−0.172
18. I am able to specify what I want to achieve and where I want to get to when I start a new task.	−0.165
40. When I work with other people in the construction of new knowledge, I am responsible for fulfilling my task within established timeframes and according to agreed procedures.	−0.161
21. I am part of social and civic groups in my community.	−0.157
35. I am able to set future short and long-term goals, and plan the steps to reach them.	−0.150
19. I think about the possible consequences before acting.	0.148
17. The fact of being in my unusual situation affects my participation and access to educational resources.	−0.121
22. My opinions are valued in my community.	0.117
11. I interact with other people for academic purposes (to spread information, share acquired knowledge, present work), using tools from my PLE such as chats, forums, social networks, blogs and wikis.	−0.104
5. I plan my work: I prioritise it, I think about the time it will take me, the resources I need and the results I hope to achieve.	

**Table 6:** Classification results ^a^.

Gender			Predicted Group Membership		Total
			Female	Male	
**Original**	**Count**	**Female**	17	3	20
		Male	9	57	66
	%	Female	85.0	15.0	100.0%
		Male	13.6	86.4	100.0%

^a^ Correctly classified 86.0% of originally grouped cases.

## Appendix A

**Table A1:** Mean, standard deviation and discrimination index obtained after administration of the socio-educational integration strategies questionnaire (SINQ).

Items	Mean	SD	R_i-t_	η2
1. I have access to computers, tablets, smartphone, etc.	3.51	4.420	0.263	0.001
2. When I find myself with a lot of information, I am able to examine it and get to what interests me.	3.33	1.175	0.334	0.004
3. I am able to organise localised information as a function of my objectives.	3.39	1.179	0.397	0.006
4. I am able to reflect before selecting the most appropriate digital tools for my learning.	3.55	1.504	0.487	0.042
5. I plan my work: I prioritise it, I think about the time it will take me, the resources I need and the results I hope to achieve.	3.46	1.435	0.452	0.001
6. I am able to manage my leisure time and for this I use tools from my Personal Learning Environment (PLE) which help me, such as agendas.	3.14	1.274	0.387	0.002
7. When I am presented with a problem, I evaluate its importance, analyse its causes and dedicate time to resolve it.	3.52	1.487	0.372	0.027
8. When I am faced with a problem and I am UNABLE to resolve it by myself, I draw on tools from my PLE that make communicating this easier.	2.81	1.472	0.395	0.006
9. I am able to maintain a conversation with other people in my own language, as much for academic purposes as social ones.	4.16	1.255	0.472	0.004
10. I am able to maintain a conversation with other people in a foreign language (Spanish), as much for academic purposes as social ones.	3.29	1.389	0.485	0.054
11. I interact with other people for academic purposes, using tools from my PLE such as chats, forums, etc.	2.91	1.469	0.463	0.035
12. I interact with other people for social purposes, using tools from my PLE such as chats and forums.	3.25	1.554	0.483	0.007
13. I share information that I know or have constructed, using tools from my PLE for this such as apps, chats, YouTube and forums.	2.61	1.563	0.280	0.016
14. That seen, heard and read online, in chats and in digital libraries, etc. has modified, clarified or broadened a concept seen in class.	2.96	1.496	0.185	0.001
15. I am able to write a digital document such as the opening of a blog or email in my own language.	3.07	1.591	0.416	0.083
16. I am able to write a digital document such as the opening to a blog or an email in a foreign language (Spanish).	2.76	1.477	0.520	0.000
17. The fact of being in an unusual situation affects my participation and access to educational resources.	3.12	1.549	0.254	0.018
18. I am able to specify what I want to achieve and where I want to get to when I start a new task.	3.71	1.232	0.650	0.004
19. Before acting I think about the possible consequences.	3.46	1.404	0.630	0.008
20. Lack of social support affects my participation in activities in my community or reduces my access to resources.	3.52	1.567	0.494	0.029
21. I am part of social or civic groups in my community.	3.01	1.657	0.218	0.003
22. My opinions are valued in my community.	3.29	1.492	0.626	0.030
23. I am able to recognise when I make a mistake and I try to correct it in the most satisfactory way possible.	3.72	1.221	0.533	0.001
24. When I experience failure I try to learn from this experience.	3.98	1.261	0.524	0.007
25. I would be able to find someone who will help me to feel better.	3.65	1.414	0.227	0.017
26. I know how to say NO when I think I have to.	3.52	1.300	0.347	0.000
27. I accept myself just as I am with my virtues and defects.	3.85	1.167	0.215	0.001
28. I identify with my community.	3.59	1.266	0.319	0.016
29. I am interested to know and to broaden my understanding of other cultures.	3.60	1.406	0.550	0.000
30. I feel as if I were owner of my community.	3.27	1.617	0.440	0.001
31. When working or communicating with other people, I listen to the different points of view and I respect them.	3.78	1.314	0.514	0.016
32. When I access web pages, blogs, forums, and I read what other people say I am respectful of their opinions.	3.41	1.404	0.560	0.004
33. When working with other members of my community I always try to help when it is in my hands to do so.	3.64	1.332	0.550	0.001
34. I like to embark upon new projects.	3.72	1.278	0.694	0.038
35. I am able to define short and long-term goals, and to plan the steps to reach them.	3.73	1.427	0.742	0.055
36. I am actively involved in completing tasks.	3.75	1.314	0.660	0.010
37. I engage in socio-recreational activity in my community.	3.54	1.142	0.703	0.020
38. My PLE (digital tools) improves my autonomous working and helps me in my student activities and to escape from my daily life.	3.52	1.412	0.751	0.000
39. I like to go deeper into certain topics and my PLE allows me to investigate them.	3.57	1.438	0.658	0.001
40. When I work with other people in the construction of knowledge, I am responsible for fulfilling my tasks within established timescales and according to the agreed procedure.	3.86	1.346	0.664	0.003

Response percentage for each scale option, mean, standard deviation and discrimination index obtained after administration of the socio-educational integration strategies questionnaire (SINQ), obtained from the present sample (*n* = 356): Mean; SD.
